# Prevalence of adverse birth outcome and associated factors among women who delivered in Hawassa town governmental health institutions, south Ethiopia, in 2017

**DOI:** 10.1186/s12978-018-0631-3

**Published:** 2018-11-26

**Authors:** Berhan Tsegaye, Andargachew Kassa

**Affiliations:** Department of Midwifery, College of Medicine and Health Sciences, Hawassa University, P.O. Box 1560, Hawassa, Ethiopia

**Keywords:** Adverse birth outcome, Women, Hawassa town, 2017

## Abstract

**Background:**

Most pregnancies are unplanned in Ethiopia. This is due to ignorance of the types and efficacy of each method of contraception they are associated with vast and unpredictable complications. Most of the time, these complications result in adverse birth outcomes. Information about prevalence of adverse birth outcome and it’s factors are relevant for designing, and initiating and intervening programs to decrease these undesirable out comes.

**Objective:**

To assess prevalence of adverse birth outcome and associated factors among women who delivered in Hawassa town governmental health institutions, south Ethiopia, in 2017.

**Method:**

We conducted institutional based cross sectional study among 580 pregnant women from december1–30/2017, by multistage systematic random sampling method in governmental health institution in Hawassa town. Data were collected through structured pre-tested, close ended and interview administered questionnaire in their post-partum period. Collected data was entered in Epi-info version 7 and analyzed using SPSS. Odds ratio with 95% confidence interval on multivariable logistic regression was computed and *P*-value< 0.05 considered as significance.

**Result:**

From a total of 580 respondents 106(18.3%) respondent’s had child related adverse birth outcome. Previous History of child related adverse pregnancy outcome 4.2 (95%,CI = 2.5–6.9), Attend at list one antenatal care visit 2.3 (95%CI = 1.1–4.3), Own cat in the house 2.2 (95%,CI = 1.3–3.7), Had any chronic disease/s 2.1(95%,CI = 1.1–4.8), Age of the mother (from 35 to 45 Years) 2.3(95%,CI = 1.1–4.8), Poor participants’ Knowledge on preconception care 3 (95%CI = 1.4–1.6) were significant predictor of adverse birth outcome in this study .

**Conclusion and recommendation:**

Prevalence of adverse birth outcome was found to be significant in the current study. Presence of Previous History of adverse pregnancy outcome, on ante-natal attendance, presence of cat in the house, presence of chronic disease/s, younger mother and Poor Knowledge of preconception care were significant predictor of adverse pregnancy outcome. Therefore it is better to give more attention on expanding preconception and antenatal care. Creating awareness about family planning methods type and efficacy for women of reproductive health is mandatory. Services, increasing health education on personal hygiene vaccination of cats. Moreover, early detecting and treatment of chronic disease.

## Plain English summary

Most pregnancy and childbirth is a joyful experience. But it sometimes end up in adverse birth outcome which disrupt the family condition leading to high individual and social costs. Adverse birth outcome in current study includes pregnancy loss/miscarriage, congenital anomaly or birth defect, preterm baby, low birth weight, perinatal death, macrosomia and intrauterine growth restriction. Most risk factors contributing to adverse birth outcome are amenable to modification. These poor reproductive life planning, substance use, abnormal pre-gestational BMI, irrational medication use, exposure to environmental toxin and contaminants, underlying chronic pre-gestational diseases, anemia and folic acid deficiency, poor regular exercise and physical activity, and presence of stress are included.

The current study use a multistage systematic random sampling technique to select the study participants. Data was collected using a validated structured pre-tested interviewer-administered questionnaire. From a total of 580 respondents 106 (18.3%) respondents had child related adverse birth outcome. Previous history of child-related adverse birth outcome, nonattendance to antenatal care owning cat in the house, having any chronic diseases, being in the age group from, and having poor knowledge about preconception care were found to be significant predictor of child-related adverse birth outcome. In conclusion: Overall prevalence of child-related abnormal birth outcome was found to be significant. Almost all predictors found as significant predictors of abnormal birth outcome are all preconception risk factors. Focusing on prevention and management of preconception risk factors through the provision of preconception care along with early engagement in maternity care continuums is highly advised.

## Background

Adverse birth outcome is a critical health issue in developing countries like Ethiopia. It resulted in many bad consequences neonatal and infant morbidity and mortality. Globally, 15 million babies are born too prematurely each year. More than a million of them die immediately after birth; many other suffer from lifelong physical, neurological, or educational disabilities [1].

According to Ethiopian Demographic and Health Survey in 2016, there was high rate of perinatal mortality. Thirty-three death per 1000 live birth was reported. It was associated with increased maternal age, primi-parity, pregnancy interval of less than 15 months and urban residence [2].

Neonatal mortality has shown less improvement than under five mortality in Ethiopia. A modeled data, indicate that Ethiopia reduced its under five mortality rate to 64/1000 live birth in 2013. It is a 69% reduction since 1990 [3]. The amazing fact here is that almost half of neonatal mortality can be prevented through tetanus toxoid vaccine, clean and skilled care at the birth, newborn resuscitation, exclusive breastfeeding, clean umbilical cord care, and management of infections in the newborns [4].

Increased number of intrauterine growth restriction is caused by low pregnancy body mass index, low short maternal stature and gestational weight. This finally leads to many low birth weight neonates in developing countries [5]. A study conducted in northern Ethiopia indicates that there were 63 deaths per 1000 live births. Among these deaths two thirds (34%) were attributable to prematurity and 21 (31%) deaths are caused by asphyxia [6].

Adverse pregnancy outcome generally increase with age. A study conducted in Nigeria, 2012 indicated that perinatal mortality, intrauterine fetal death, and neonatal death increased with age [7]. As evidenced by many research findings, adverse pregnancy outcomes had both child and maternal short term and long term undesirable effects. A study conducted in North West Ethiopia, 2016 showed that Low birth weight and preterm delivery increased the risk of asthma at age of 7 [8]. A study conducted in Nepal, 2013 showed that birth order of the child, with which wantedness is inextricably linked, has more powerful and pervasive effects, with first-born and second-born children being much less likely to show adverse effects [8]. Experiencing intimate partner violence during pregnancy has been associated with women’s increased risk of antepartum hemorrhage, and perinatal death. [9] A study conducted in Boricha district of Sidama zone, southern Ethiopia,2014, indicates that having had a neonatal, infant and/or child deaths was associated with symptoms of depression at 14-year follow-up [10].

The achievement of sustainable development goal is highly associated with declining of neonatal death. Though, there are studies on the various forms of adverse birth outcomes particularly in developing countries and few part of Ethiopia, there is limited data on the overall adverse birth outcome at country level specifically at Hawassa town governmental health institution. Therefore, the aim of this study is to determine the prevalence and associated factor of adverse birth outcomes among women who give birth in Governmental health institution in Hawassa town.

## Methods and materials

### Study area and period

Hawassa city administration is a capital city of southern nation nationality people region and located 275 km South from Addis Ababa. Hawassa city in which 359,358 people are living according to city’s Health Department Estimation in 2017, has been structured by 7 urban sub-cities collectively have 21 kebeles and 1 Rural sub-city includes 11 kebeles. The city has 83 public and private health institutions. In detail, these are one public referral and teaching hospital, one public general hospital, four private primary hospitals, 9 Public Health Centers, 17 Public Health Posts and 51 Private Clinics.

There are about a total of 852 health professionals working in the randomly selected PHIs of the city. Together with about 600 health professionals working in Hawassa University Comprehensive Specialized Hospital, the number reached1452.

Hawassa University Comprehensive Specialized Hospital is the largest hospital in southern Ethiopia with more than 300 beds which renders service to both the people in the region and other people coming from the neighboring region. The hospital was established in 2004/05, provides specialized services and comprehensive health services (prevention, treatment and research). The outpatient department consists of 17 rooms and inpatient service consists of five main departments. The average number of patients flow at the outpatient department is more than 200 people per day. The Hawassa University Comprehensive Specialized Hospital and Adare General Hospital are the only public health hospitals providing comprehensive essential obstetric care in the City. The remaining nine PHC are giving Basic essential obstetric care. In the past 6 months of the year 2009, there were a total of 4780 deliveries reported from Hawassa University Comprehensive Specialized Hospital (2073), Adare General public health officer (2022), Adare primary health care (313), Millennium primary health care (311), and Tilte primary health care (61).

### Study variables

The dependent variable was the ‘overall adverse birth outcomes ’. Whereas, the independent variables tested in this study included factors grouped into five categories. The first category includes socio-demographic factors such as age, parity, educational status, religion, occupation, partner’s occupation, residence, and family size. The next category is the mother’s current obstetric condition. This obstetric condition includes parity, gravidity, antenatal care attendance, gestation, pregnancy complication, birth order, and mother’s age at current pregnancy.

The third category is the presence of pre-existing medical conditions. This included conditions such as diabetes mellitus, hypertension, cardiac disease, HIV/AIDS, anemia, malaria, and oral health status. The preconception risk factors occurring before pregnancy such as the woman’s pregnancy intention, psychoactive substance use, coffee consumption, pre-gestational BMI, periodontal disease, medication use, previous history of, the use of traditional medicine, environmental exposure, pre-gestational disease, preconception folic acid supplementation use status, presence of external stressors, familial/genetic history, medical insurance status, physical abuse, women’s preconception health/care knowledge, inter-pregnancy interval, regular exercise, and uptake of preconception counseling. The last or fifth category is health facility-related factors. This included factors such as availability of health facility, distance, and cost of service.

#### Operational definition

‘Adverse birth outcome” in this study implies the presence of at least one or more of the following conditions in the current pregnancies. These include fetal loss/miscarriage, low birth weight, congenital anomaly, and preterm labour. Thus, if the mothers admitted to the labour ward gave birth to a baby with a congenital anomaly, low birth weight, preterm labour, small for gestational age, perinatal death, macrosomia then the these were labeled as “mothers with a child-related adverse birth outcome. Those who gave normal live birth, without the above mentioned abnormal birth outcome , were labeled as “mothers with normal child related pregnancy outcome”.

**Perinatal mortality**: Is defined as deaths inside uterus (Macerated and fresh stillbirths) and early neonatal deaths (first week of life).Perinatal mortality rate is the sum of both of them per 1000 total deliveries [11].

**Miscarriage**: Is the natural death of an embryo or fetus before it is able to survive independently [12].

**Low birth weight**: The weight of the newborn < 2.5 kg [13].

**Preterm labour**: Preterm labor is defined as regular contractions of the uterus resulting in changes in the cervix that start before 37 weeks of pregnancy [14].

**Macrosomia**: Macrosomia is defined as birth-weight over 4000 g irrespective of gestational age [15].

**Congenital anomaly**: A congenital anomaly defined as any abnormality of physical structure found at birth or during the first few weeks of life; or any irreversible condition exiting in a child before birth in which there is sufficient deviation in the usual number, size, shape, location of any part, organ, cell to warrant its designation as abnormal [16, 17].

### Sample size and sampling procedures

At first stage, out of the 11 public health institutions, five public health institutions were selected using simple random sampling technique. At the second stage, we applied a systematic random sampling method to identify and include all the study participants. The calculated sampling interval (K) was 1.2. Based on the finding we consecutively recruited the study subject’s i.e. using SRS up until the minimum sample size obtained. This is in accord with the assumption that deliveries in nature happened randomly. The samples were taken proportionate to the number of expected deliveries from each selected PHIs. All participants included in the study were all consented verbally to participate in the study. Excluded out of the study were mothers with a twin pregnancy.

### Study design and population

A cross-sectional study was conducted among women giving birth in public health institution of Hawassa City Administration. The source populations were all pregnant women living in the Hawassa City Administration. Whereas, the study population were those laboring mothers who attended the randomly selected public health institutions for delivery service during the study period.

This study applied a multistage sampling technique to identify a total of 580 study participants.

The sample size required for this study was determined using the formula for estimating single population proportion. Since there is no research (as per the investigators best systematic literature search) no published article reported the overall prevalence of abnormal birth outcome, and also the proportion of client’s attitude towards preconception care. Thus a prevalence value of 0.5 was assumed to calculate the minimum sample size. On the basis of this assumption the minimum possible sample size (n) sufficient enough to meet objectives of the study. The sample size determination considered the following assumptions. This included a 95% confidence interval (CI), a margin of error (α = 0.05), population correction factor, a design effect of 2 and 10% non-response rate.

### Data collection and analysis

The principal investigator developed the instrument used in this study by referring to various literatures. The instrument is developed based on the objectives of the study. It was primarily designed to measure all the variables listed in the preceding two paragraphs. The instrument was a validated interviewer-administered questionnaire. The instrument was first developed in English and translated into Amharic and then back to English to check the accuracy. Before the actual data collection, the questionnaire was tested on 10% of health care providers working in Public health Institutions of a nearby town called Shashemene.

Every woman who came to deliver in the selected primary health institutions during the data collection period was all interviewed. In addition to the interview, the data collectors abstracted clinical data by reviewing the mother and the babies’ medical records. They also measured and confirmed the baby’s birth weight, and conducted selected examinations for which they get additional training. The Data was collected by 5 BSC female midwives and 5 BSC female nurses after one-day Training about informed consent, techniques of interviewing, data collection procedures, and different sections of the questionnaire. Two health officers were assigned as Supervisors for the data collectors.

Overall supervision also made by the Principal Investigators. All collected questionnaires were rechecked for completeness and coded. Then these data were entered and cleaned using SPSS version 20 for analysis. Bivariable and multivariable logistic regression was employed to identify an association between the independent predictors and the outcome variable i.e. “child-related adverse birth outcome”. Those factors found with their *P*-value ≤0.20 in the bi-variable logistic regression model were fitted into the multivariable logistic regression model to control the effect of confounding variables. Multivariable analysis using standard logistic regression technique was done to evaluate the independent effect of each covariate on ‘child-related adverse birth outcome *’* by controlling the effect of others. Variable having *P*-value of less than 0.05 in the multivariable logistic regression analysis was considered as significantly associated factors of ‘child-related adverse birth outcome ’. The adjusted odds ratios with the 95% Confidence Intervals (CI) were reported. Before the actual logistic regression analysis was done, the necessary assumption of logistic regression model was checked by using Hosmer-Lemeshow test of goodness of fit which has a chi-square distribution. For further analysis, descriptive statistics like frequencies and cross tabulation were performed. Tables and bar chart were used to present the findings of the study.

## Result

### Socio-demographic characteristics

From a total of 580 women delivered at governmental health institutions in Hawassa town, all participated fully in this study making a response rate of 100%. The mean age of respondents is 25.63 + 4.9 years. Majorities 492(84.6%) were in the age range of 20–34 years. Most of the study participants 560(96.6%) were married. More than half, 325(56%), of participants attended in elementary education. Whereas, only few (8.3%) attended college or university level education.

Most of the study participants (68.8%) were housewives and the greater proportion (78.6%) had a family size less than five person per a household. From the total of 580 study participants, most 375(64.7%) of the study participants were living in urban (Table 1). Almost equal number of respondent's partner (50.5%) use family planing methods (see Table 2).

**Table 1:** Socio-demographic characteristics women in postpartum period in Hawassa university referral hospital, southern Ethiopia, 2017(*n* = 580)

Variables	Categories	N	Percent (%)
AGE	< 20	45	7.8
	20–34	492	84.6
	35–49	43	7.4
Religion	protestant	293	50.5
	Orthodox	157	27.1
	Muslim	87	15
	catholic	8	1.4
	others	13	2.2
Marital status	Married	560	96.6
	Single	20	3.4
Ethnicity	sidama	232	40
	Amhara	42	7.2
	Gurauge	46	7.9
	Oromo	115	19.8
	wolayta	106	18.3
	silte	39	6.8
educational status	no formal education	110	19
	Primary education (1–8 grade)	325	56
	Secondary education (9–12 grade)	97	16.7
	Tertiary (college or university)	48	8.3
Women’s occupation	housewife	399	68.8
	private business	87	15
	daily worker	14	2.4
	salaried employed	61	10.5
	Student	19	3.3
Husband occupation	Farmer	153	26.4
	Daily worker	41	7.1
	private business	237	40.9
	salaried employed	124	21.4
	Student	3	0.5
	others	22	3.8
Monthly income	< 1000 ETB	174	30
	1001–2000 ETB	119	20
	2001–3675 ETB	142	25
	> 3675 ETB	145	24
Total family size	< 5	456	78.6
	5–6	89	15.3
	> 6	35	6
residence	urban	375	64.7
	rural	150	25.9
	peri-urban	55	9.4

keyETB = ethiopian birr(1US$ = 27ETB)

**Table 2:** Reproductive characteristics of women in postpartum period in Hawassa university referral hospital, southern Ethiopia, 2017(*n* = 580)

Variables	Categories	Frequency	Percent
Did you or your partner use FP?	Yes	293	50.5
	No	287	49.5
what type of FP do you use? (*n* = 293)	coc	38	6.6
	Depo	189	32.6
	Implanon	23	4
	iucd	34	5.9
	Natural	8	1.4
	no FP	287	49.5
Do you plan this pregnancy?	Yes	520	89.7
	no	60	10.3
Gestational age of the current pregnancy?	preterm	58	10
	term	519	89.5
	post term	3	0.5
Did the mother had at least 1 ANC visit?	yes	521	89.8
	no	59	10.2
mode of delivery	SVD	461	9.5
	instrumental	11	1.9
	CS	108	18.6
sex of the current baby?	male	330	56.9
	female	250	43.1
future pregnancy intention	up to 6moth	1	0.2
	7–12 months	2	0.3
	13–24 months	9	1.6
	25–36 months	8	1.4
	after 3 years	132	22.8
	undecided	375	64.7
	I don’t want any more	531	9.1

*KEY=FP* family planning, *IUCD* intrauterine contraceptive device, *SVD* spontaneous vertex delivery, *CS* cesarean section

### Maternal related adverse pregnancy outcome

**Fig. 1:**
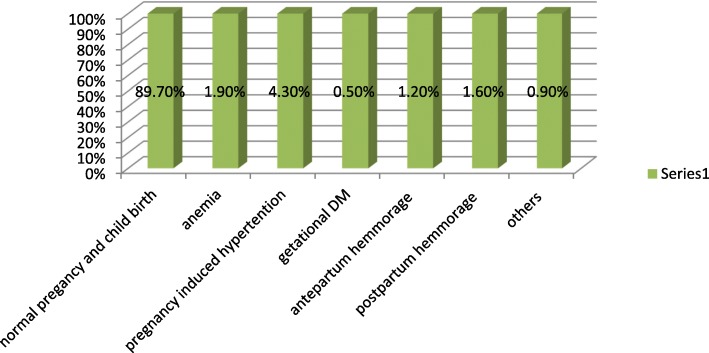
Maternal related adverse obstetrics complications of women in Governmental health institutions in Hawassa town, southern Ethiopia, 2017. (*n*=580)

Pregnancy induced hpertension which account(4.3%) is the observed predominant maernal related adverse outcome (Fig. 1).

### Prevalence of adverse pregnancy outcomes

The current study reveal that 472(81.3%) births are normal live births, while the remaining 108(18.6%) births were births with child related adverse birth outcomes. The current study identified 7 types of abnormal birth outcome. Out of which 67 (11.6%) was low birth weight and the perinatal death was 16(2.7%). The magnitude of congenital anomaly was 6(1%). The study also identified two types of abnormal birth outcome among 33 (5%) of the newborns (Table 3).

**Table 3:** Magnitudes and types of adverse pregnancy outcome related to the child among deliveries attended in public health institutions of Hawassa, Southern Ethiopia 2017

S.N	List of adverse pregnancy outcomes		Frequency	Percent
1	Perinatal death	No	564	97.2
		Yes	16	2.7
		Total	580	100.0
3	Preterm baby or birth (PB)	No	559	96.4
		Yes	21	3.6
		Total	580	100.0
4	Congenital anomaly	No	574	99.0
		Yes	6	1.0
		Total	580	100.0
5	Macrosomia	No	517	89.13
		Yes	63	10.86
		Total	580	100.0
6	IUGR	No	577	99.5
		Yes	3	.5
		Total	580	100.0
7	Low Birth Weight	No	513	88.4
		Yes	67	11.6
		Total	580	100.0
8	Number of APOs observed in a new birth	No APO observed	474	81.7
		Only 1 APO observed	73	12.6
		Two (2) types of APO observed	33	5.7
		Total	580	100.0
9	Over all APO related to the child	No APO	474	81.7
		APO	106	18.3
		Total	580	100.0

KEY

*LBW* low birth weight

### Predictors of overall adverse pregnancy outcomes identified

This study identified six statistically significant predictors of adverse pregnancy outcomes related to the child. The chance of developing an abnormal birth outcome among mothers with a previous history of child-related abnormal birth outcomes was by four times higher than those mothers who didn’t have the history (AOR = 4.2, 95% CI: 2.5–6.9). Compared to mothers within the age range 20–34 years, those mothers within the age range 35–45 had two-fold higher odds of giving birth to a baby with APO than those mothers who aged 20–34 years (AOR = 2.3, 95% CI: 1.1–4.2). Non attendance to antenatal care, in contrast to attending at least one ANC, showed an increased odd of APO (AOR = 2.2, 95% CI: 1.1–4.3). The likely hood of giving a baby with an adverse pregnancy outcome was by two times higher among those mothers owned a cat in their house (AOR = 2.2, 95% CI: 1.3–3.7) and those who had any chronic diseases(AOR = 2.2, 95% CI: 1.1–4.8). The analysis also revealed that the odds of giving birth to a baby with APO are by three-fold higher among mother with poor preconception care/health knowledge than those with good preconception care/health knowledge (AOR = 3.0, 95% CI: 1.4–6.6) (Tables 3 and 4).

**Table 4:** Predictors of child related adverse pregnancy outcome from backward stepwise (Wald) logistic regression, in Governmental health institutions, Hawassa Ethiopia, 2017

Predictors		Adverse pregnancy outcome (APO)		COR[95%CI]	AOR[95%CI]
		No APO	Yes APO		
Previous History of child related APO	Yes	87	48	2.3(1.3–4.1) *	4.2(2.5–6.9)***
	No	387	58	1^§^	1
Previous History of maternal APO	Yes	42	19	3.7(2.4–5.8) ***	1.7(0.8–3.6)
	No	432	87	1	1
Age of the Mother	20–34 Years	411	81	1	1
	18–19 Years	27	16	1.3(0.6–2.7)	1.5(0.6–3.7)
	35–45 Years	36	9	3.0(1.6–5.8) **	2.3(1.1–4.8) *
Attend at list one ANC visit	Not attended	40	19	2.4(1.3–4.3) *	2.2(1.1–4.3)*
	Attended	434	87	1	1
Duration b/n the current & previous pregnancies in months	1st pregnancy	229	38	1	1
	< 12 Months	21	14	4.0(1.9–8.6) ***	2.3 (0.9–5.4)
	12–17 Months	29	8	1.7(0.7–3.9)	1.3(0.5–3.3)
	18–23 Months	66	17	1.6(0.8–2.9)	1.6(0.8–3.3)
	> 24 Months	129	29	1.4(0.8–2.3)	0.7(0.4–1.3)
Anemia in current pregnancy	Yes	60	21	1.7(0.9–3.0)	1.5(0.8–2.8)
	No	414	85	1	1
Own cat in the house?	Yes	162	55	1.8(1.2–2.7) *	2.2(1.3–3.7) **
	No	312	51	1	1
Had any chronic disease/s	Yes	34	16	2.3(1.2–4.4)*	2.2(1.1–4.8)*
	No	440	90	1	1
Mothers’ PCC knowledge	Good Knowledge	105	10	1	1
	Fair Knowledge	139	25	1.9(0.9–4.1)	2.2(0.9–5.5)
	Poor Knowledge	230	71	3.2(1.6–6.5) **	3.0(1.4–6.6) *
Monthly income	≤1000.0birr	133	41	1.8(1.1–3.3)*	1.4(0.7–2.7)
	1001.0-2000.0birr	99	20	1.2(0.6–2.3)	1.1(0.5–2.4)
	2001.0 -3675.0 birr	118	24	1.2(0.6–2.3)	1.2(0.6–2.4)
	≥ 3676 birr	124	21	1	1

Key 1 = references group, * = PV < 0.05, ** = PV < 0.001, *** = PV < 0.0001, *CI* confidence interval, *COR* crude odds ratio, *AOR* adjusted odds ratio, *ANC* antenatal care, *PCC* preconception care, *ETB* Ethiopian birr(1$ = 27ETB)

## Discussion

This study aimed to assess the overall prevalence of abnormal birth outcome and preconception risk factors contributing to child-related abnormal birth outcome among deliveries attended in primary health institutions of Hawassa. All women approached to be included in the study attended the study. This makes the response rate 100%. The overall prevalence of adverse birth outcome in the current study was found to be 18.6% (95%CI = 0.3–38.6%). Whereas, the types and proportions of the specific abnormal birth outcome identified in this study were; low birth weight 67(11.6%), macrosomia 26(4.5%), preterm birth 21(3.6%), congenital anomaly 6(1.0%), intrauterine growth restriction 3(0.5%), and perinatal death 16 (2.7%).

The magnitude of abnormal birth outcome in the current study is significant. Compared to other similar study findings reported from Ethiopia [18, 19], the prevalence of the overall abnormal birth outcome identified in this study is low. The possible explanation for this discrepancy may be attributable to socioeconomic variations and the difference in the quality of maternal health service. In addition the variation with regard to nutritional and cultural practices may also contribute to the observed differences.

Moreover, since most of the current study participants were urban residence their level of knowledge and utilization of health service might be the other reason to the observed lower abnormal birth outcome. Except for the mother’s non-attendance to ante natal care, all of the five predictors identified in the current studies are preconception risk factors contributing to the development of child-related abnormal birth outcome. Similar to two studies in Gondar [18]and Dessie [20], the odds of giving birth to a baby with abnormal birth outcome was found higher than those mothers who attended antenatal care (AOR = 2.2, 95% CI: 1.1–4.2). In Ethiopia, non-attendance to antenatal care is one of the most important public health problems which preconception care intervention can solve. There is an evidence that the odd of uptake of antenatal care is higher among mothers who get preconception care than those who are not getting the service [21]. In Ethiopia, for the past two decades, the antenatal care service uptake remained very low. This is evident from those national demographic health survey reported an increase from 27% in 2000 to 62% in 2016 [2]. This sluggish increment in antenatal care attendance may be accelerated by the introduction of preconception service in the countries health care system. As one study denoted, despite the world health organization’s recommendation, preconception care is not yet formally and officially introduced in the Ethiopian health care system [22].

The chance of developing an abnormal birth outcome among mothers with a previous history of child-related abnormal birth outcome was by four times higher than those mothers who had not had the history (AOR = 4.1, 95% CI:2.5–6.8). Having had a child with abnormal birth outcome, in the previous pregnancy/ies, is one of the most important predictors of all the five predictors identified in this study. A similar study conducted in Shire town of North Ethiopia showed the fact those mothers with the previous history of child related abnormal birth outcome are at greater risk of giving birth to a baby with [19]. There is well-established evidence that previous history of abnormal birth outcome is a risk factor to the occurrence of abnormal birth outcome in subsequent pregnancies. Significant proportion 135(23.3%) of the study participants, in the current study, had a history of child-related adverse pregnancy outcomes. Through preconception care the healthcare, the provider can screen out such risk factors prior to pregnancy. This preventive preconception care intervention is one of the recommended evidence-based practice to solve the occurrence of similar abnormal birth outcome in subsequent pregnancies. One study showed the need and uptake of preconception care are higher among women with previous history abnormal birth outcome [23].

Similarly, the odd of giving birth to a baby with abnormal birth outcome was by 2 times higher among women with chronic diseases than those who didn’t have(AOR = 2.3, 95% CI: 1.1–4.9). This finding is consistent with a study conducted at Dessie [20]. Chronic diseases such as diabetes, hypertension, asthma, epilepsy, depression are well-known risk factors to abnormal birth outcome [24, 25]. Early identification and management of chronic diseases including mental health, prior to conception, is proven to bring a normal pregnancy outcome [4]. Delaying pregnancy until the control of the underlying chronic diseases is one of the objectives of preconception care. This is managed by using preconception family planning service and effective medical care. Nevertheless, there is high the unmet need for family planning among this group of people. Those women with chronic medical conditions are at risk of abnormal birth outcome demanding preconception care [26].There is evidence that advanced maternal age is a risk factor for the occurrences of both maternal and child related abnormal birth outcome s [27]. In line with these evidence, this study revealed a twofold higher odds of abnormal birth outcome among women in the age group 35–45 Years than those in the age group 20-34 years (AOR = 2.395% CI: 1.1–4.8). The co-occurrence of chronic diseases with pregnancy is common. This is one of the cases that complicate the outcome of pregnancies. Though, an advanced maternal age is one of the non-modifiable risk factors for pregnancy, early enrolment of women in preconception care may help couples to make a wise reproductive plan [26]. Those women with a poor knowledge of preconception health/care were 3 times (AOR = .3.0,95%, CI: 1.4–6.6) more likely to have an abnormal birth outcome than those who had good knowledge. A community-based study conducted at Adet, North Ethiopia, showed poor knowledge among the vast majority of the women [28]). If the woman has appropriate knowledge of preconception health and care, she can seek or easily attend to the preconception care service. This can help her to easily identify and effectively control the underlying risk condition that leads to abnormal birth outcome. Health care providers participated in one research reported that poor awareness or knowledge of preconception health and care as a barrier to the uptake and delivery of preconception care [28]. Another systematic study that investigated the reasons why women are not using preconception care reported poor PCC knowledge and awareness as a barrier to preconception care uptake [29].

The last predictor identified by the current study showed that those women who have cat in their house had two times higher odds of abnormal birth outcome than those who didn’t have (AOR = 2.3, 95% CI: 1.4–3.8). The possible explanation for this result may be toxoplasmosis infection that uses cats as a reservoir can lead leads adverse birth outcome like premature birth and low birth weight. Studies conducted at different areas of Ethiopia showed high prevalence of Sero-prevalence of Toxoplasma gondii (T.gondii) antibodies for IgM among pregnant women [30–32]. This studies reported that contact with cat or cat ownership as factors that increased the odds of women’s to T.gondii infection and recommended avoidance of contact with cats and avoidance of raw and under cooked foods [31–33]).

In general this study identified five preconception risk factors or that increased the mothers odds of giving birth to a baby with abnormal birth outcome. Nevertheless, his study is not without limitation. Some of participants of the study may not remember some of the questions. This may lead to underreporting of the findings reported in this study. For some of the variables, such as previous history of abnormal birth outcomes, this study relied on the study participants verbal response. This study never included abnormal birth outcomes s happening at an and post pregnancy period. This include abnormal birth outcome such as miscourage, pregnancy loss, neonatal and infant death. As a cross sectional study this study do didn’t determined causal relationship and also temporal relationship. Despite this study, might tried to focus on preconception risk factors contributing to abnormal birth outcomes. To the best of authors best systematic search, there are not published articles reported preconception risk factors contributing to abnormal birth outcomes in Ethiopia.

## Conclusion and recommendation

The prevalence of the overall adverse birth outcome is significant. Previous History of child related abnormal birth outcomes, women’s poor knowledge of preconception health/care, having any chronic diseases, advanced maternal age (35–45 years), and having a cat in the house were all preconception risk factors that increased the likelihood of developing child related abnormal birth outcomes. Non attendance to antenatal care was the other factor that increased the odds of the occurrence of abnormal birth outcomes. Provision of preconception and inter-conception care is the best approach to the identification and early management of risk factors to abnormal birth outcomes. The continuums of the maternal and child health care services are incomplete without the inclusion of PCC. Adverse pregnancy outcome reduction strategies should consider early identification and management of the preconception risk factors. The preconception care should include educating all eligible or reproductive-aged couples about preconception care/health. Creating awareness about family planning methods type and efficacy for women of reproductive health is mandatory.

## Abbreviations

ABO: Adverse birth outcome
AOR: Adjusted odds ratios
CI: Confidence interval
COR: Crudes odds ratios
CSA: Central Statistical Agency
EDHS: Ethiopian Demographic and Health survey
PCC: Preconception care
